# Innovative Strategies in 3D Bioprinting for Spinal Cord Injury Repair

**DOI:** 10.3390/ijms25179592

**Published:** 2024-09-04

**Authors:** Daniel Youngsuk Kim, Yanting Liu, Gyubin Kim, Seong Bae An, Inbo Han

**Affiliations:** 1 Research Competency Milestones Program (RECOMP), School of Medicine, CHA University, Seongnam-si 13488, Republic of Korea; dkim304@chauniv.ac.kr; 2 Department of Medicine, School of Medicine, CHA University, Seongnam-si 13496, Republic of Korea; 3 Department of Neurosurgery, CHA Bundang Medical Center, CHA University, Seongnam-si 13496, Republic of Korea; yantingliu02@gmail.com (Y.L.); gyubink06@gmail.com (G.K.); anseongbae@gmail.com (S.B.A.)

**Keywords:** spinal cord injury, bioprinting, stem cells, conductive material, neurotrophic factors, multi-channel

## Abstract

Spinal cord injury (SCI) is a catastrophic condition that disrupts neurons within the spinal cord, leading to severe motor and sensory deficits. While current treatments can alleviate pain, they do not promote neural regeneration or functional recovery. Three-dimensional (3D) bioprinting offers promising solutions for SCI repair by enabling the creation of complex neural tissue constructs. This review provides a comprehensive overview of 3D bioprinting techniques, bioinks, and stem cell applications in SCI repair. Additionally, it highlights recent advancements in 3D bioprinted scaffolds, including the integration of conductive materials, the incorporation of bioactive molecules like neurotrophic factors, drugs, and exosomes, and the design of innovative structures such as multi-channel and axial scaffolds. These innovative strategies in 3D bioprinting can offer a comprehensive approach to optimizing the spinal cord microenvironment, advancing SCI repair. This review highlights a comprehensive understanding of the current state of 3D bioprinting in SCI repair, offering insights into future directions in the field of regenerative medicine.

## 1. Introduction

**Figure 1:**
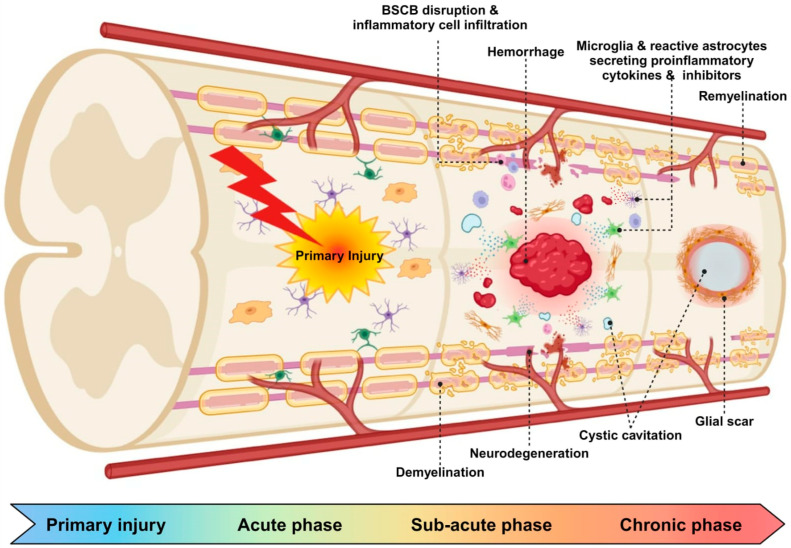
Pathophysiological progression of spinal cord injury. This figure illustrates the pathophysiological progression of SCI from primary injury through to the chronic phase. The process begins with a primary injury, represented by a lightning bolt, which causes immediate damage to the spinal cord tissue. Following this, the acute phase involves blood–spinal cord barrier disruption, inflammatory cell infiltration, and hemorrhage. As the injury progresses into the sub-acute phase, microglia and reactive astrocytes secrete proinflammatory cytokines and inhibitors, contributing to demyelination and neurodegeneration. The chronic phase is characterized by ongoing pathological changes, including cystic cavitation, glial scar formation, and limited attempts at remyelination.

Spinal cord injury (SCI) is a debilitating condition marked by the disruption of neurons and axons within the spinal cord, resulting in substantial motor and sensory deficits below the injury site, commonly due to traumatic events [1]. During the primary injury phase, trauma causes immediate mechanical damage. This sets off cellular and molecular responses that break down neural tissue and disrupt the barrier between the blood and the spinal cord. The secondary injury phase, characterized by inflammation, excitotoxicity, and oxidative stress, exacerbates the initial damage, further impeding neural regeneration and repair [2] (Figure 1). The complexity of SCI poses significant challenges to recovery due to the complex structure of the spinal cord, the limited regenerative capacity, and the inhibitory environment created by glial scar formation and myelin-associated inhibitors [3].

Contemporary interventions for SCI, such as surgical decompression and stabilization, anti-inflammatory drugs, and rehabilitation, mainly aim to mitigate secondary damage and promote recovery [4]. The primary objective of methylprednisolone, the initial medication approved by the Food and Drug Administration (FDA) for SCI, is to impede lipid peroxidation, manage inflammation, and function as a free radical scavenger to safeguard the spinal cord–blood barrier and enhance blood circulation to the affected region [5,6]. However, its clinical benefits remain controversial due to significant adverse effects, such as increased risks of urinary tract, respiratory, and wound infections [7]. Surgical treatments, such as laminectomy and fusion, aim to relieve pressure on the spinal cord and stabilize the spine [8]. Despite these interventions, current clinical treatment methods offer only a marginal improvement in outcomes and do not facilitate functional regeneration of the injured spinal cord [9].

Tissue engineering for SCI has emerged as a promising field that aims to overcome the limitations of conventional therapeutic approaches [10]. This interdisciplinary field combines principles of biology, engineering, and material science principles to develop bioengineered constructs that support neural regeneration and functional recovery. Within the realm of SCI, tissue engineering mainly centers on creating scaffolds that can imitate the natural extracellular matrix (ECM) of the spinal cord. This replication facilitates the survival, proliferation, and differentiation of neural cells [11].

The introduction of three-dimensional (3D) bioprinting has resulted in substantial advances in tissue engineering, particularly for complicated structures such as the spinal cord [12]. 3D bioprinting enables the precise fabrication of scaffolds with intricate geometries and microarchitectures that closely mimic the natural structure of neural tissues. This technology allows for the layer-by-layer construction of scaffolds customized to fit the specific dimensions and needs of the injured spinal cord [13,14]. One of the primary benefits of 3D bioprinting is the ability to insert living cells directly into the scaffold during printing, resulting in a more biomimetic environment that promotes cell survival and integration [15].

The objective of this review is to provide a comprehensive overview of 3D bioprinting techniques, bioinks, and stem cell applications. Additionally, we also review recent advancements in 3D bioprinting, including the integration of conductive materials, the development of biofunctional scaffolds incorporating neurotrophic factors, drugs, and exosomes, and the design of innovative structures such as multi-channel and axial scaffolds, all of which are aimed at neural regeneration and functional recovery in SCI.

## 2. 3D Bioprinting Technologies for SCI Repair

### 2.1. Overview of 3D Bioprinting Techniques and Bioinks

3D bioprinting has emerged as a pivotal technology in SCI repair, offering the ability to create complex, biomimetic scaffolds that support neural regeneration and re-establish functional neural circuits by promoting cell proliferation and differentiation [16], facilitating neurotrophic factor release [17,18], and controlling inflammation [19,20,21].

**Figure 2:**
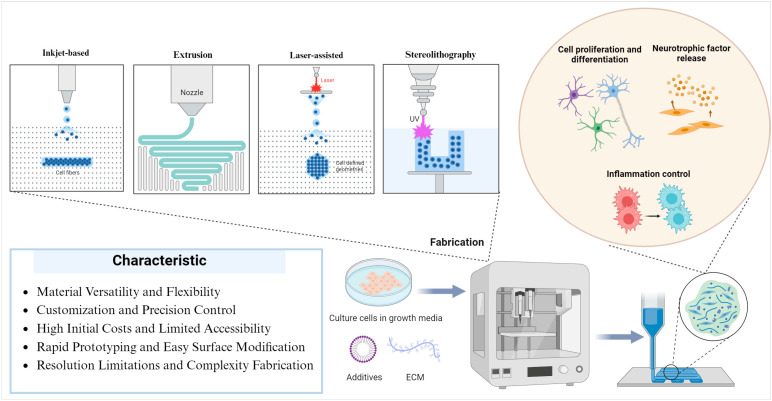
Bioprinting techniques and applications in tissue engineering. This figure illustrates various bioprinting techniques and their applications in tissue engineering and regenerative medicine. The bioprinting methods shown include inkjet, extrusion, laser-assisted, and stereolithography. The cells, depicted as small pink structures in a culture dish, are prepared for the fabrication stage, where they are combined with ECM components and additives before being introduced into a bioprinter. The bioprinter, represented in the middle, combines various biological materials and controls their precise placement to construct complex tissue structures. It depicts the process of culturing cells in growth media post-fabrication, emphasizing applications in cell proliferation, differentiation, neurotrophic factor release, and inflammation control. For the inflammation control, the red cells symbolize M1 macrophages related to pro-inflammatory responses. The blue cells represent M2 macrophages associated with anti-inflammatory and tissue repair functions.

The primary techniques employed in 3D bioprinting include extrusion-based, inkjet-based, laser-assisted bioprinting, and stereolithography, each with particular advantages customized to different parts of scaffold production [18,22,23]. The most often employed technology, extrusion-based bioprinting, enables the controlled deposition of highly viscous bioinks, making it ideal for creating structured scaffolds with embedded cells [24,25]. Inkjet-based bioprinting, noted for its extreme precision in cell placement, enables the systematic organization of cellular components. However, it is constrained by the necessity for low-viscosity bioinks, which may impair scaffold stability [26,27]. Laser-assisted bioprinting offers exceptional resolution and cell viability, although it is less widely adopted due to its complexity and cost [28]. Stereolithography, which uses a vat polymerization process, allows for the fabrication of highly detailed and complicated 3D structures with high resolution. However, it poses obstacles, such as the possible damage of photoinitiators to living cells and a time-consuming process [29] (Figure 2). Table 1 provides the four prominent 3D bioprinting technologies: extrusion-based, laser-assisted, inkjet-based, and stereolithography. Each column corresponds to a different bioprinting method and describes critical aspects such as applicable bioinks, precision, printability, primary applications, advantages, disadvantages, and recent innovations associated with each technology. It also outlines how each method caters to specific requirements in tissue engineering.

The bioinks used in 3D bioprinting are critical to the success of SCI treatment. These bioinks typically consist of natural or synthetic hydrogels combined with living cells and bioactive molecules, forming a water-swollen polymer network that provides a hydrated environment similar to the natural ECM [30]. Natural hydrogels, such as alginate, gelatin, and hyaluronic acid (HA), are preferred for their biocompatibility and ability to mimic the ECM, efficiently encapsulating cells and supporting survival [31]. On the other hand, synthetic polymers have variable mechanical properties, improving the scaffolds’ structural integrity [32]. Recent advancements have led to the development of composite bioinks that incorporate conductive materials, and decellularized extracellular matrix components, significantly boosting the bioactivity and therapeutic potential of the printed constructs. Table 2 summarizes the various material categories employed in biomedical engineering, particularly tissue engineering and regenerative medicine. It categorizes materials into classes and describes their main characteristics, applications, and associated issues. Each entry describes how these materials contribute to tissue engineering—from enhancing cell adhesion and proliferation to promoting vascularization and dynamic responsiveness in tissue constructs—while highlighting specific limitations such as mechanical stability, immunogenicity, and degradation rates. This table serves as a resource for creating biologically compatible scaffolds and devices.

### 2.2. Role of Stem Cells in 3D Bioprinting for SCI Repair

Stem cells play an important role in 3D bioprinting for SCI repair, with the potential to replace injured brain tissue and restore lost functions [23]. Neural stem cells (NSCs) are especially important in this setting due to their ability to differentiate into various neural cell types, including neurons, astrocytes, and oligodendrocytes [54]. Integrating stem cells into 3D bioprinted scaffolds has demonstrated potential for improving their survival, proliferation, and directed differentiation within the injury site. Several studies have demonstrated the efficacy of different types of stem cells, including NSCs, induced pluripotent stem cells (iPSCs)-derived progenitors, and ectomesenchymal stem cells (EMSCs) in 3D bioprinted constructs [16,17,21]. These cells are often embedded within hydrogels that mimic the ECM, providing a supportive environment for their maturation and integration into the host tissue.

NSCs are multipotent stem cells that can differentiate into neurons, astrocytes, and oligodendrocytes, all of which are important cell types in the central nervous system [55]. The application of NSCs in SCI repair has shown promising results, particularly when these cells are incorporated into 3D bioprinted scaffolds [56]. Within these scaffolds, NSCs have demonstrated an enhanced ability to promote axonal regeneration and reduce the formation of inhibitory glial scars, advancing neural repair in SCI. Studies have shown that NSC-laden scaffolds can support the survival and differentiation of these cells at the injury site, resulting to significant improvements in motor and sensory function in animal models of SCI [37,57,58]. These scaffolds provide a structured environment that promotes cell survival and guides the regenerating axons, helping to reestablish neural connections essential for functional recovery [59].

iPSCs provide an additional layer of potential, primarily because they can be generated from a patient’s cells, lowering the risk of immune rejection and avoiding ethical concerns associated with embryonic stem cells [60]. iPSCs can be reprogrammed into NPCs or directly into other neural cell types, which can then be embedded into bioprinted scaffolds. These cells have been shown to enhance axonal regeneration and promote the formation of new neural circuits in SCI models [61]. Furthermore, when delivered through bioprinted scaffolds, iPSC-derived NPCs show increased integration with host tissue, contributing to improved motor function and reduced secondary damage processes like inflammation and scarring [62].

EMSCs derived from the cranial neural crest are ideal for 3D bioprinting because they can differentiate into a variety of cell types, including neurons and glial cells, which are critical for SCI repair. The precision of 3D bioprinting allows for the creation of scaffolds that mimic the natural environment of these cells, enhancing their growth and differentiation [63]. Specialized hydrogels provide a supportive matrix that maintains structural integrity and facilitates the sustained release of essential growth factors such as brain-derived neurotrophic factor (BDNF), encouraging the EMSCs to develop into functional neural tissue.

One study demonstrated that the transplanting of human umbilical cord mesenchymal stem cells (MSCs) onto collagen/silk fibroin scaffolds could effectively promote nerve regeneration and the recovery of neurological function after SCI [64]. Another advanced research investigated the use of 3D-printed collagen/silk fibroin scaffolds infused with the secretome from human umbilical MSCs to improve neurological function after SCI in rats. This approach addresses issues such as low cell survival and ethical concerns associated with direct MSC transplantation. The secretome, a collection of proteins secreted by MSCs, provides a more stable and clinically applicable alternative. The study discovered that these scaffolds accelerated nerve fiber regeneration, improved motor function recovery, and facilitated synaptic connections at the injury site [65]. Recently, researchers explored the development of a 3D printable hydrogel composed of sodium alginate and matrigel to facilitate the differentiation of EMSCs into neurons [66]. The novel sodium alginate/matrigel scaffold enhanced neuronal differentiation efficiency compared to traditional 2D cultures, offering a promising biomaterial for neuron regeneration and SCI treatment. The hydrogel’s drug-releasing capabilities were investigated, and results revealed a sustained release of BDNF, which promotes neuronal growth and differentiation.

## 3. Innovative Scaffold Designs for Enhanced Regeneration

In tissue engineering, hydrogels create an aqueous environment similar to the natural ECM, supporting cell growth and function [30]. Hydrogels, on the other hand, are unable to conduct electrical signals, which are required for interacting cells, facilitating neural stem cell differentiation and regeneration, preventing scar tissue formation, and enhancing biological function. To promote neural repair, conductive materials such as conductive polymers and nanomaterials are incorporated into hydrogels to create conductive scaffolds that promote signal transmission and tissue regeneration [67,68].

### 3.1. Enhancing Neural Repair with Conductive Scaffolds

Polypyrrole (PPy) is a widely utilized conductive polymer for neural tissue engineering due to its high conductivity, stability, ease of synthesis, and cellular biocompatibility [69]. Entezari et al. explored the effectiveness of 3D-printed polycaprolactone/PPy conductive scaffolds in promoting the differentiation of human olfactory EMSCs into Schwann cell-like phenotypes. These scaffolds provided an environment conducive to the differentiation of olfactory EMSCs, resulting in increased secretion of nerve growth factors, such as nerve growth factor (NGF) and BDNF, and enhanced neurite outgrowth [70].

Poly(3,4-ethylenedioxythiophene) (PEDOT), derived from polythiophene, is well-known for its exceptional chemical stability and electrical conductivity, making it an alternative to PPy for developing electrically responsive scaffolds. Furthermore, PEDOT’s ability to form stable and high-quality films with excellent adhesion to various substrates further supports its use in 3D bioprinted scaffolds [71,72]. However, due to PEDOT’s low solubility, dopants are frequently used to improve its water solubility and stability. Gao and Song et al. were motivated to synthesize PEDOT with alternative dopants to overcome these limitations while maintaining its desirable properties and developed a PEDOT-based NSCs-laden conductive composite scaffold using 3D bioprinting. Doping PEDOT with sulfonated lignin improved the conductivity of the scaffold by nearly tenfold and reduced impedance, thereby supporting high NSC survival rates and increased differentiation both in vitro and in vivo [73]. Similarly, Song et al. doped PEDOT with chondroitin sulfate to improve its water solubility, electric properties, and biocompatibility. Furthermore, the axially stacked NSC-laden 3D bioprinted scaffold guided the neurite outgrowth [74]. When implanted into a SCI rat model, these conductive scaffolds delivered NSCs effectively, promoted the formation of new nerve tissue, regenerated nerve fibers, and reduced glial scarring, thus facilitating hindlimb functional recovery [75].

Incorporating conductive nanomaterials such as carbon nanotubes (CNTs) into scaffolds improves their electrical properties, contributing to developing advanced neural tissue constructs with superior tensile strength, thermal stability, and conductivity [50]. Lee et al. developed 3D-printed conductive scaffolds using polyethylene glycol diacrylate (PEGDA) polymer and multi-walled CNTs. These scaffolds achieved intricate microarchitectures and controlled porosity using stereolithography and improved electrical properties that significantly increased the proliferation and differentiation of NSCs seeded on the 3D scaffolds [49].

Kiyotake et al. integrated gold nanorods (GNRs) into HA/gelatin hydrogels to improve conductivity. Nevertheless, the rise was not as substantial as that of gold nanoparticles because greater swelling of the hydrogel was observed in GNR hydrogels. Furthermore, GNR hydrogels exhibited a stiffness that was 2.9 times greater and a yield stress that was 2.4 times higher. Significantly, the NSCs cultivated on GNR scaffolds did not demonstrate early neural differentiation, emphasizing the necessity for additional investigation into integrating GNR hydrogels with electrical stimulation [76]. Although not involving 3D bioprinting technology, conductive hydrogels incorporating gold nanoparticles with MXene demonstrated higher free radical-scavenging rates, improved conductivity, and reduced bacterial survival. When these hydrogels were combined with NSCs and subjected to electrical stimulation, they significantly enhanced NSC proliferation, differentiation into nerve cells, and the formation of synaptic connections between neurons [77].

According to clinical investigations, electrical stimulation has a good potential for restoring neurologic function and improving motor and autonomic function in patients with SCI [78]. Furthermore, integrating electrical stimulation with conductive scaffolds is a promising technique for SCI repair because it promotes axonal regeneration by activating cyclic adenosine monophosphate, which triggers critical molecular pathways for nerve regeneration [79]. Conductive scaffolds incorporating materials like PPy or CNTs may benefit from including electrical stimulation [80,81], and implementing this strategy within 3D bioprinted scaffolds should further increase therapeutic outcomes in SCI repair.

### 3.2. Biofunctionalization of Scaffolds with Neurotrophic Factors, Drugs, and Exosomes

Neurotrophic factors are polypeptide growth factors that activate intracellular signaling pathways by targeting specific receptors, which are necessary for neuronal survival, differentiation, and growth. Integrating neurotrophic factors into biocompatible scaffolds in SCI research is increasingly recognized for enhancing the regenerative microenvironment, promoting neuronal survival, axonal regeneration, and synaptic plasticity [17,82,83]. BDNF is essential for nervous system development and provides neuroprotective advantages following damage by activating the tyrosine kinase receptor B, which promotes axonal regeneration and neuronal connectivity [84,85]. Liu et al. developed a 3D collagen and chitosan scaffold using low-temperature extrusion 3D printing technology and integrated BDNF during printing. This method resulted in a more effective and prolonged BDNF release than post-printing absorption, leading to enhanced locomotor function, nerve fiber regeneration, synaptic connections, and remyelination in SCI models [86]. Similarly, NGF is a neurotrophic protein essential for the growth and survival of neural cells [84]. Lee et al. fabricated a 3D biomimetic scaffold by incorporating poly(lactic-co-glycolic acid) (PLGA) nanoparticles encapsulating NGF into a hydrogel scaffold. The NGF-embedded scaffold effectively sustained drug release from core-shell PLGA nanoparticles, leading to enhanced PC-12 cell proliferation, increased neurite length, and guided neurite extension [87]. While 3D bioprinted scaffolds are typically static, four-dimensional (4D) printing introduces the element of time, enabling controlled, time-dependent biochemical distribution [88]. For example, Chiang et al. employed 4D spatiotemporal control for the dynamic release of neurotrophin-3 (NT-3), supporting the differentiation and migration of endogenous NSCs [89].

Conventional ways of administering exogenous biomolecules, such as systemic injection or oral administration, frequently require increased efficacy due to low drug stability and rapid clearance, necessitating higher doses that may cause undesirable side effects. Drug-loaded 3D bioprinted scaffolds solve these problems by allowing for the localized and controlled release of bioactive compounds while providing structural support and guidance for tissue regeneration [90]. Liu et al. developed a 3D bioprinted scaffold using a bioink composed of gelatin methacryloyl (GelMA) and acrylate β-cyclodextrin, which provided an optimal environment for NSCs through its high water content and uniformly porous structure. Additionally, the scaffold loaded with an O-GlcNAc transferase (OGT) inhibitor demonstrated a stepwise release over 72 h, effectively promoting neuronal differentiation of NSCs by inhibiting the Notch signaling pathway [91]. Another study utilized oxymatrine, a quinazoline alkaloid with anti-inflammatory, antioxidant, and anti-fibrotic properties, in 3D microfiber scaffolds through electrospinning technology. These scaffolds demonstrated sustained release for up to 30 days, effectively suppressing inflammation, recruiting endogenous NSCs, and encouraging neuronal differentiation while inhibiting glial scar formation at the lesion site [92].

Exosomes, known for their strong biocompatibility, low immunogenicity, excellent stability, and modifiability, play a crucial role in the intercellular transport of neuroprotective substances. Engineered exosomes can be modified to carry small molecules or nucleic acids, making them practical for targeted drug delivery in SCI treatment [93,94]. Shang et al. developed 3D bioprinted scaffolds encapsulating PTEN-interfering siRNAs-loaded exosomes. These exosomes, derived from bone marrow mesenchymal stromal cells (BMSCs), alleviated the inflammatory response, reduced scar formation, and supported neuroregeneration. Furthermore, PTEN-interfering siRNAs were encapsulated within the exosomes to achieve PTEN inhibition, enhancing downstream mTOR phosphorylation and promoting axonal regeneration. As a result, these PTEN-interfering siRNAs encapsulated exosome-loaded scaffolds facilitated nerve connectivity and signal transmission within the damaged spinal cord [95]. Furthermore, plant-derived exosomes, which are naturally occurring lipid bilayer extracellular vesicles with antiviral, anti-inflammatory, and antitumor properties, have been utilized in 3D bioprinting. Due to their plant origin, these exosomes have a reduced risk of triggering immunogenic reactions compared to animal-derived or synthetic nanoparticles [96]. Wang et al. developed 3D bioprinted scaffolds incorporating isoliquiritin encapsulated within plant-derived exosomes from Lycium barbarum (ISL@PE). ISL@PE exhibited controlled release for up to 16 days, even at low pH levels. Furthermore, ISL@PE reduced the expression of inducible nitric oxide synthase and reactive oxygen species, polarized the microglia from the M1 to the M2 phenotype, and inhibited inflammation by upregulating phosphorylated AKT expression. As a result, the ISL@PE-laden 3D-printed scaffold facilitated nerve regeneration, decreased scar tissue development, and reduced inflammatory responses in SCI rat models [97].

Beyond the neurotrophic factors and biomolecules mentioned previously, scaffolds have also been designed to incorporate glial cell line-derived neurotrophic factor, ciliary neurotrophic factor, NT-3, and curcumin [98,99,100,101,102]. Advances in 3D bioprinting will enable the development of even more sophisticated scaffolds that integrate these elements. Furthermore, when combined with conductive hydrogels, these advanced scaffolds could synergistically promote tissue repair after spinal cord injury by modulating the immune response and enhancing the growth of myelinated axons [103].

### 3.3. Multi-Channel Conduits and Axial Structured Scaffolds: Promoting Neuronal Connectivity

Multi-channel conduits are designed to emulate the complicated architecture of the spinal cord, providing multiple pathways that align with the natural direction of axonal growth. Scaffold channel design is critical for allowing cell infiltration, axon growth, and nerve regeneration because the architecture directly affects cellular morphology, function, motility, attachment, and orientation [104]. Microchannels larger than 450 μm in scaffolds have been found to reduce axonal regeneration [105], while those with diameters around 150–200 μm are most effective in guiding axons in a linear direction [106]. Joung et al. developed a 3D multi-channel scaffold using microextrusion-based 3D printing with 150 μm diameter channels composed of alginate and methylcellulose. Spinal neuronal and oligodendrocyte progenitor cells (sNPCs and OPCs) were precisely positioned within the scaffold using a point-dispensing method. The sNPCs differentiated into neurons that project axons across the scaffold, while OPCs grew into oligodendrocytes that myelinate these axons, forming a neural relay network. Two weeks after printing, functional neural activity was found, confirming that the bioprinted neurons survived, differentiated, and matured [39]. Lee et al. used extrusion 3D bioprinting to create a multi-channel conduit composed of collagen and alginate. The 3D multi-channel scaffolds were designed to longitudinally align Schwann cells and endothelial cells within the channels, facilitating axon regrowth and migration along neovessels. The study also found that increasing the number of channels led to improved outcomes. A 9-channel conduit outperformed a 5-channel conduit, showing more outstanding biocompatibility, cell proliferation, neuronal regeneration, remyelination, inflammation reduction, and angiogenesis [40]. A 3D bioprinted PEGDA-GelMA scaffold with 200 μm diameter channels was constructed utilizing microscale continuous projection printing and a UV light source. The printing process can generate customized rodent spinal cords in 1.6 s and scale them to human size. In contrast to standard grid structures, the scaffold’s structure matches the spinal cord’s inherent architecture. Channels in the white matter linearly guided axonal regeneration, while the grey matter, which is generally devoid of axonal projections, was engineered as a solid block to increase the mechanical strength of the scaffold. When loaded with NPCs, these scaffolds support axon regeneration and synapse formation [107]. The benefits of these multi-channel designs can be further enhanced by incorporating conductive materials and neurotrophic factors [108,109,110]. Combined with 3D bioprinting, these enhancements could synergistically improve scaffold performance in supporting neural regeneration and facilitating functional recovery in SCI.

3D bioprinting has also been used to create axial-structured scaffolds that resemble densely packed bundles of nerve fibers. Yang et al. combined 3D bioprinted scaffolds made of GelMA and hyaluronic acid methacrylate (HAMA) with NSCs to construct living nerve-like fibers with interconnected porosity. This structure facilitated the transport of nutrients and waste, which is critical for the long-term survival of encapsulated cells and creates an optimal environment for NSCs. These scaffolds enabled neuronal relay formation and overall neural regeneration by enhancing the circumstances at the defect site through immune regulation, angiogenesis, neurogenesis, and neural circuit remodeling [111]. In an advanced approach, Li et al. utilized coaxial 3D extrusion printing to develop hierarchically structured scaffolds with dual-network hydrogels. The inner layer, composed of HA derivatives and N-cadherin-modified sodium alginate, provided sustained mechanical support and promoted NSC migration and neuronal differentiation by down-regulating the level of β-catenin in the cytoplasm. The outer layer, consisting of gelatin/cellulose nanofiber hydrogel loaded with metalloporphyrin, rapidly released the reactive species scavenger to protect endogenous NSCs in the early stages of SCI. These hierarchically structured scaffolds enhanced neural network formation, inhibited glial scar formation, and reduced collagen deposition in SCI rats [112]. 

Table 3 provides a comprehensive overview of the integration of innovative techniques into 3D bioprinted scaffolds for SCI repair, detailing the bioinks, cell types, printing methods, experimental models, innovative technologies, and outcomes associated with each scaffold.

## 4. Challenges and Future Perspectives

### 4.1. Technical and Biological Challenges in 3D Bioprinting for SCI

The application of 3D printing in spinal cord regeneration faces several significant challenges despite its promising potential in regenerative medicine. One of the key challenges is selecting and developing bioinks, which are the components utilized in the printing process. These bioinks must be biocompatible and possess sufficient mechanical strength and stability to support the growth of neural tissue. However, many current bioinks are derived from non-human sources, which can come with the risk of immunogenicity and infection. Furthermore, it is challenging for these materials to meet the mechanical, rheological, and biological requirements for efficient spinal cord regeneration.

Current bioprinting technologies have failed to master the ability to accurately replicate the spinal cord’s complicated neuronal networks and the unique composition of its grey and white matter. This precision is critical because it influences the alignment and distribution of numerous cell types within the bioprinted scaffolds, which are required for functional recovery. The challenge is not only in scaffold design but also in maintaining high cell viability and ensuring that cells are precisely distributed within these structures to mimic the natural environment of the spinal cord. In parallel, the materials used for bioprinting these scaffolds must support essential cellular functions such as adhesion, proliferation, and differentiation. These materials must also be biocompatible and biodegradable to avoid long-term adverse effects on the body. However, finding or developing materials that meet all these criteria remains a formidable challenge. Most existing materials result in scaffolds that are overly simplistic and fail to recreate the native 3D structure and biochemical environment of the spinal cord tissue. This deficiency reduces the likelihood of effective nerve regeneration and functional integration. Another critical aspect is the integration of bioprinted scaffolds with the host tissue, which relies heavily on effective vascularization. To guarantee appropriate nutrient and oxygen delivery to the implanted cells, tissue-engineered implants must have a strong blood supply for long-term survival and performance. Without this, the chance of cell death and implant failure rises significantly [113]. Ensuring vascularization within bioprinted structures is thus a pivotal area of focus, as it is essential for the survival and overall efficacy of the treatment.

Furthermore, the potential immunogenicity of bioprinted materials can provoke significant immune responses, leading to inflammation and possibly the rejection of the scaffold. Managing immunogenicity and ensuring that bioprinted structures do not elicit negative immune responses are critical for promoting healing and regeneration. These immune problems complicate the development of viable bioprinted therapeutics for SCI, requiring a delicate balance between material characteristics and immune compatibility [114]. Finally, translating 3D bioprinting from experimental models to clinical applications involves navigating a labyrinth of regulatory and ethical considerations. Ensuring the safety and efficacy of bioprinted products requires rigorous clinical trials and regulatory approval processes. Furthermore, ethical concerns, particularly those related to the source and type of cells used, must be addressed in order to properly advance these technologies.

### 4.2. Future Trends in 3D Bioprinting-Based Therapies

As the field of 3D bioprinting for SCI repair continues to advance, numerous emerging themes have the potential to enhance treatment outcomes significantly. One of the most promising trends, as stated in Section 3, is the integration of novel approaches into 3D bioprinted scaffolds. Future scaffolds are likely to incorporate conductive materials, bioactive compounds such as neurotrophic factors, drugs, and exosomes, as well as innovative structural designs that mimic the intricate architecture of the spinal cord. This integration aims to produce a synergistic impact that improves neuronal regeneration and functional recovery.

Another notable development is the growing emphasis on personalized medicine. Advances in bioprinting technology enable the creation of patient-specific scaffolds that are tailored to the unique anatomical and pathological aspects of each individual’s damage. This personalized technique may improve therapy efficacy by ensuring that the bioprinted scaffold is perfectly tailored to the patient’s spinal cord geometry and lesion site.

## 5. Summary

3D bioprinting has emerged as a critical technology in SCI repair, utilizing appropriate bioinks and stem cell applications to produce scaffolds that closely replicate the native architecture of the spinal cord. Integrating innovative strategies with 3D bioprinting offers a multimodal approach to improving the spinal cord microenvironment for SCI repair. This review underscores advancements in improving electrical conductivity, biofunctionalization, and structural features to enhance therapeutic efficacy. Incorporating conductive materials and bioactive molecules, such as neurotrophic factors, drugs, and exosomes, has been shown to improve neural differentiation and facilitate axonal growth, thereby supporting functional recovery. Multi-channel conduits and axially structured scaffolds effectively align with the natural direction of axonal growth and enhance neural network formation. Despite these advances, significant challenges remain, such as duplicating the complicated anatomy of the spinal cord, developing biocompatible and functioning bioinks, and establishing adequate vascularization within the printed constructions. Addressing materials’ biocompatibility, integrating bioprinted tissues with host tissues, and regulating the immune response to implanted constructions will be critical. Future research should prioritize optimizing these technologies for clinical applications, emphasizing safety, scalability, and efficacy. Interdisciplinary collaboration among biologists, neuroscientists, and clinicians is required to bring these advances from the lab to clinical practice.

## Figures and Tables

**Table 1:** Comparative characteristics of 3D bioprinting technologies.

Characteristic	Extrusion-Based	Inkjet-Based	Laser-Assisted	Stereolithography
Applicable Bioinks	Broad spectrum: hydrogels, thermoplastics, and composites	Best for low-viscosity, aqueous solutions	Limited to photopolymerizable materials	Restricted to UV-sensitive resins and specific photopolymers
Precision	Medium: Ideal for building foundational structures	High: Great for detailed surface features, less so for complex 3D forms	Very High: Excellent for intricate micro-architectures	Extremely High: Capable of nano-scale precision
Printability	Robust for large, continuous structures.	Efficient in producing high-resolution patterns, suitable for microfluidics	Excellent for creating fine and complex vascular networks	Best for achieving complex geometries with high accuracy
Primary Applications	- Scaffold construction - Soft tissue engineering - Bone and cartilage regeneration	- Skin substitutes - Tissue chips for drug testing - Simple organoids	- Microvasculature fabrication - Nerve regeneration constructs - Precision cartilage implants	- Dental applications - Complex tissue models - High-precision implants
Advantages	- Versatile with material types - Supports multi-material printing - Continuous material deposition ideal for large constructs	- High printing speed - Low material waste - Capable of printing gradients - Precise droplet control	- Ultra-high resolution - Minimal cell damage due to non-contact process - Ideal for high-detail tissue layers	- Unmatched precision - Capable of printing complex internal structures - Smooth surface finish, critical for tissue interfaces
Disadvantages	- Lower resolution compared to laser methods - Potential for high shear stress on cells - Slower build times	- Limited to less viscous materials - Lower structural integrity in larger prints - Potential for cell damage during droplet ejection	- Applied in nerve regeneration and wound healing - Supports angiogenesis and axonal regrowth	- High cost and complexity - Limited bioink selection - UV exposure can adversely affect cell viability
Recent Innovations	- Incorporation of real-time mechanical feedback for better structural accuracy - Development of shear-thinning bioinks to reduce cell damage	- Advancement in droplet-based systems to improve cell viability - Introduction of on-demand printing with fewer material constraints	- Use of multi-photon polymerization for even finer details - Integration of biocompatible photo-initiators to expand material choices	- Development of hybrid resins that allow for faster curing without compromising biocompatibility - Improvement in software algorithms for optimizing print paths
References	[24,25]	[26,27]	[23,28]	[23,29]

**Table 2:** Characteristics of material categories in biomedical engineering.

Material Category	Key Features	Applications	Challenges	References
Natural Polymers	Collagen: Abundant, supports cell adhesion, low immunogenicity, facilitates tissue integration.	- Enhances axonal guidance and reduces scar formation. - Ideal for scaffolds in neural tissue repair.	- Requires crosslinking for stability. - Rapid enzymatic degradation.	[33,34]
	Gelatin and GelMA: Easy modification, supports 3D bioprinting, promotes cell proliferation.	- Used in 3D bioprinting of scaffolds. - Facilitates neural differentiation.	- Mechanical weakness; needs combination with stronger polymers. - Fast degradation.	[35,36]
	Hyaluronic Acid: Natural hydrating agent, promotes cell migration and proliferation.	- Encourages axonal growth and neural tissue repair. - Useful in hydrogels for spinal injury treatment.	- Weak structural integrity. - Rapid breakdown under physiological conditions.	[36,37,38]
Polysaccharide-based	Alginate: Forms stable hydrogels, highly biocompatible, easily modified for drug delivery.	- Applied in controlled release systems for drug delivery. - Supports 3D cell culture.	- Limited mechanical properties. - Requires modification for cell adhesion.	[36,39,40]
Protein-based	Fibrin: Excellent compatibility, promotes vascularization, fast degradation.	- Useful in early-stage wound healing and tissue repair. - Supports cell adhesion and proliferation.	- Rapid breakdown, potentially leading to instability. - Careful handling required to avoid rapid clotting.	[36,41,42]
Polysaccharide-based	Chitosan: Biodegradable, antimicrobial, and supports hemostasis.	- Applied in nerve regeneration and wound healing. - Supports angiogenesis and axonal regrowth.	- Variable mechanical properties. - High immunogenicity in some formulations.	[36,43]
Synthetic Polymers	Polycaprolactone: Strong, long-lasting, shape-memory capabilities.	- Ideal for long-term implants and load-bearing applications. - Supports tissue engineering scaffolds.	- Slow degradation rate, potential persistence in the body. - Requires surface modification for cell adhesion.	[36,44]
	Polyethylene Glycol: Versatile, non-toxic, customizable degradation.	- Used in drug delivery systems. - Reduces inflammation and oxidative stress.	- Lacks intrinsic biological activity. - Often needs to be combined with bioactive materials.	[36,45]
	Poly(lactic-co-glycolic acid): FDA-approved, controlled degradation, supports drug release.	- Popular for drug delivery vehicles. - Enhances tissue regeneration.	- Acidic degradation by-products may cause inflammation. - Requires precise control of degradation rate.	[46]
Elastomers	Poly(glycerol sebacate): Elastic, suitable for dynamic environments, promotes vascularization.	- Applied in soft tissue engineering, such as cardiac and neural tissues. - Biodegradable.	- Limited cell adhesion due to hydrophobicity. - Requires surface modification.	[47]
Biocomposites	Decellularized Extracellular Matrix: Mimics native tissue, supports cell attachment.	- Enhances tissue integration in vivo. - Promotes cell-specific differentiation.	- Complex production process. - Potential variability in composition and residual immunogenicity.	[48]
Nanomaterials	Carbon-based (CNTs): Conductive, promotes cell growth, excellent mechanical properties.	- Useful in developing conductive scaffolds for neural applications. - Enhances electrical signal conduction.	- Potential cytotoxicity. - Difficult to functionalize and process.	[49,50]
Thermoresponsive Polymers	Methylcellulose: Injectable, biocompatible, forms gels at body temperature.	- Ideal for minimally invasive cell delivery systems. - Supports stem cell transplantation.	- Poor mechanical strength. - Needs reinforcement with other materials for structural applications.	[39,51]
Biodegradable Plastics	Polyhydroxyalkanoates: Biodegradable, microbial origin, tunable properties.	- Sustainable material for tissue engineering. - Useful in slow-release drug delivery.	- High production cost. - Variability in mechanical properties based on the microbial source.	[52]
Advanced Proteins	Silk Fibroin: High tensile strength, biocompatible, slow-degrading, versatile processing.	- Applied in long-term implants and tissue scaffolding. - Supports cell attachment and growth.	- Complex processing and purification required. - Potential immunogenicity if not properly processed.	[33,53]
Self-assembling Materials	Self-assembling Peptides: Customizable, forms nanofibers mimicking the extracellular matrix.	- Useful in targeted drug delivery and regenerative medicine. - Supports neural tissue formation.	- High cost and synthesis complexity. - Potential immunogenicity depending on peptide sequence.	[36]

**Table 3:** Summary of included studies on the integration of innovative techniques into 3D bioprinted scaffolds for SCI repair.

Bioink	Cell Type	Printing Method	In Vitro/In Vivo	Innovative Technologies	Outcomes	References
Enhancing conductivity						
Polycaprolactone and PPy	Olfactory EMSC	Extrusion	In vitro	Enhancing conductivity by PPy	- PPy improved the conductivity of 3D-printed scaffolds. - The conductive scaffolds promoted the differentiation of MSCs into Schwann cell-like phenotypes, enhancing the secretion of nerve growth factors and neurite outgrowth.	[70]
GelMA, HAMA, and PEDOT: sulfonated lignin	NSC	Extrusion	In vivo	Enhancing conductivity by PEDOT	- PEDOT improved the conductivity of scaffolds by nearly tenfold and reduced impedance. - The 3D bioprinted scaffolds supported high survival rates and increased differentiation of encapsulated NSCs in vitro and in vivo.	[73]
GelMA, PEGDA, PEDOT: chondroitin sulfate methacrylate, and TA	NSC	Extrusion	In vitro	Enhancing conductivity by PEDOT	- Doping PEDOT with chondroitin sulfate improved its water-solubility, electrical properties, and biocompatibility. - The axially stacked 3D bioprinted scaffold tguided neurite outgrowth.	[74]
GelMA, PEGDA, PEDOT: chondroitin sulfate methacrylate, and TA	NSC	Extrusion	In vivo	Enhancing conductivity by PEDOT	- The conductive 3D bioprinted scaffold showed high conductivity, shape fidelity, shear-thinning, and self-healing properties. - The 3D bioprinted scaffold enhanced neuronal differentiation and locomotor function recovery in SCI rats.	[75]
PEGDA and CNTs	NSC	Stereolithography	In vitro	Enhancing conductivity by CNTs	- Stereolithography enabled intricate microarchitectures and controlled porosity. - Improving electrical properties by CNTs enhanced the proliferation and differentiation of NSCs seeded on the 3D-printed scaffolds.	[49]
Pentenoate-functionalized HA & gelatin and GNR	NSC	Extrusion	In vitro	Enhancing conductivity by GNR	- GNRs enhance conductivity and stiffness. - GNRs are not sufficient alone to drive NSC’s differentiation without the electrical stimulation.	[76]
Biofunctionalization (Neurotrophic Factors, Drugs, and Exosomes)						
Collagen and chitosan	NSC	Extrusion	In vitro (In vivo—without NSC laden)	Adding BDNF	- Integrating BDNF during the 3D printing process prolonged release of BDNF. - The 3D bioprinted scaffold improved locomotor function, nerve fiber regeneration, synaptic connections, and remyelination in SCI rats.	[86]
PEGDA with NGF-loaded PLGA nanoparticles	PC12 cell	Stereolithography	In vitro	Adding NGF-loaded PLGA nanoparticles	- Incorporating PLGA nanoparticles encapsulating BSA and NGF enabled sustained release of bioactive factors. - The nanoparticle loaded scaffold significantly increased neurite length and effectively guided neurite extension.	[87]
GelMA and acrylate β-cyclodextrin	NSC	Extrusion	In vivo	Adding OGT inhibitor	- OGT inhibitor-laden 3D bioprinted scaffolds allow for localized, controlled release of bioactive molecules. - The scaffolds promoted neuronal differentiation by inhibiting the Notch signaling pathway in SCI rats.	[91]
GelMA	PC12 cell	Extrusion	In vitro (In vivo—without PC12 cell laden)	Adding PTEN-interfering siRNAs-loaded exosomes	- The 3D-printed scaffold encapsulated PTEN-interfering siRNAs-loaded exosomes. - siRNAs inhibited PTEN, which in turn enhanced mTOR phosphorylation and facilitated axonal regeneration.	[95]
GelMA	NSC	Extrusion	In vitro (In vivo—without NSC laden)	Adding plant-derived exosomes loaded with isoliquiritin	- The 3D-printed scaffold incorporated isoliquiritin encapsulated within plant-derived exosomes. - The scaffold controlled drug release and reduced inflammation by modulating microglial polarization and increasing phosphorylated AKT expression.	[97]
Multi-Channel Conduits and Axially Structured Scaffolds						
Matrigel, gelatin/fibrin, and GelMA	NPC and OPC	Extrusion	In vitro	Multi-Channel Conduits	- The 3D multichannel scaffold with 150 μm diameter channels incorporated sNPCs and OPCs. - The sNPCs differentiated into neurons and OPCs matured into oligodendrocytes, creating a neural relay system.	[39]
Alginate and collagen	Schwann cell and endothelial cell	Extrusion	In vivo	Multi-Channel Conduits	- 3D multichannel scaffold, designed to align Schwann cells and endothelial cells for axon regrowth and migration. - Increasing the number of channels led to improved outcomes.	[40]
PEGDA and GelMA	NPC	Microscale continuous projection	In vivo	Multi-Channel Conduits	- The 3D biomimetic scaffold with 200 μm diameter channels was created through continuous projection printing, allowing fast and high-resolution customized rodent spinal cords. - The scaffold enhanced mechanical properties and cellular attachment, leading to functional recovery.	[107]
GelMA and HAMA	NSC	Extrusion	In vivo	Axially Structured Scaffolds	- The axially structured 3D bioprinted scaffold was designed to resemble densely arranged bundles of nerve fibers. - The scaffold enhanced neural relay formation and overall neural regeneration in SCI rats.	[111]
Inner layer: HA derivatives and N-cadherin modified sodium alginate Outer layer: gelatin/cellulose nanofiber	NSC	Extrusion	In vitro (In vivo—without NSC laden)	Axially Structured Scaffolds	- Hierarchically structured scaffolds with dual-network hydrogels, where the inner layer supports NSC migration and neuronal differentiation, and the outer layer protects NSCs by releasing reactive species scavengers. - The scaffold improved neural network formation, inhibited glial scar formation, and reduced collagen deposition in SCI rats.	[112]

## Abbreviations

SCI = Spinal Cord Injury. FDA = Food and Drug Administration. ECM = Extracellular Matrix. 3D = Three-Dimensional. HA = Hyaluronic Acid. NSCs = Neural Stem Cells. iPSCs = Induced Pluripotent Stem Cells. EMSCs = Ectomesenchymal Stem Cells. BDNF = Brain-Derived Neurotrophic Factor. MSCs = Mesenchymal Stem Cells. PPy = Polypyrrole. NGF = Nerve Growth Factor. PEDOT = Poly(3:4-ethylenedioxythiophene). CNTs = Carbon Nanotubes. PEGDA = Polyethylene Glycol Diacrylate. GNRs= Gold Nanorods. PLGA = Poly(lactic-co-glycolic acid). 4D = Four-Dimensional. NT-3 = Neurotrophin-3. GelMA = Gelatin Methacryloyl. OGT = O-GlcNAc Transferase. BMSCs = Bone Marrow Mesenchymal Stromal Cells. ISL@PE = Plant-Derived Exosomes from Lycium Barbarum. sNPCs = Spinal Neural Progenitor Cells. OPCs = Oligodendrocyte Progenitor Cells. HAMA = Hyaluronic Acid Methacrylate.

## References

[1] Eckert M.J., Martin M.J. (2017). Trauma: Spinal Cord Injury. Surg. Clin. N. Am..

[2] Alizadeh A., Dyck S.M., Karimi-Abdolrezaee S. (2019). Traumatic Spinal Cord Injury: An Overview of Pathophysiology, Models and Acute Injury Mechanisms. Front. Neurol..

[3] Ueno M., Yamashita T. (2008). Strategies for regenerating injured axons after spinal cord injury—Insights from brain development. Biologics.

[4] Lewis N.E., Tabarestani T.Q., Cellini B.R., Zhang N., Marrotte E.J., Wang H., Laskowitz D.T., Abd-El-Barr M.M., Faw T.D. (2022). Effect of Acute Physical Interventions on Pathophysiology and Recovery After Spinal Cord Injury: A Comprehensive Review of the Literature. Neurospine.

[5] Takami T., Shimokawa N., Parthiban J., Zileli M., Ali S. (2020). Pharmacologic and Regenerative Cell Therapy for Spinal Cord Injury: WFNS Spine Committee Recommendations. Neurospine.

[6] Gadot R., Smith D.N., Prablek M., Grochmal J.K., Fuentes A., Ropper A.E. (2022). Established and Emerging Therapies in Acute Spinal Cord Injury. Neurospine.

[7] Hurlbert R.J. (2014). Methylprednisolone for the treatment of acute spinal cord injury: Point. Neurosurgery.

[8] Tabarestani T.Q., Lewis N.E., Kelly-Hedrick M., Zhang N., Cellini B.R., Marrotte E.J., Williamson T., Wang H., Laskowitz D.T., Faw T.D. (2022). Surgical Considerations to Improve Recovery in Acute Spinal Cord Injury. Neurospine.

[9] Cristante A.F., Barros Filho T.E., Marcon R.M., Letaif O.B., Rocha I.D. (2012). Therapeutic approaches for spinal cord injury. Clinics.

[10] Jia Z., Li W. (2024). Nanosystems-enabled regenerative strategies for spinal cord Injury: Recent advances and future prospects. Mater. Des..

[11] da Silva V.A., Bobotis B.C., Correia F.F., Lima-Vasconcellos T.H., Chiarantin G.M.D., De La Vega L., Lombello C.B., Willerth S.M., Malmonge S.M., Paschon V. (2023). The Impact of Biomaterial Surface Properties on Engineering Neural Tissue for Spinal Cord Regeneration. Int. J. Mol. Sci..

[12] Saini G., Segaran N., Mayer J.L., Saini A., Albadawi H., Oklu R. (2021). Applications of 3D Bioprinting in Tissue Engineering and Regenerative Medicine. J. Clin. Med..

[13] Gu Z., Fu J., Lin H., He Y. (2020). Development of 3D bioprinting: From printing methods to biomedical applications. Asian J. Pharm. Sci..

[14] Xie Z., Gao M., Lobo A.O., Webster T.J. (2020). 3D Bioprinting in Tissue Engineering for Medical Applications: The Classic and the Hybrid. Polymers.

[15] Persaud A., Maus A., Strait L., Zhu D. (2022). 3D Bioprinting with Live Cells. Eng. Regen..

[16] Zarepour A., Hooshmand S., Gökmen A., Zarrabi A., Mostafavi E. (2021). Spinal Cord Injury Management through the Combination of Stem Cells and Implantable 3D Bioprinted Platforms. Cells.

[17] Lu D.Z., Yang Y., Zhang P.P., Ma Z.J., Li W.T., Song Y., Feng H.Y., Yu W.Q., Ren F.C., Li T. (2022). Development and Application of Three-Dimensional Bioprinting Scaffold in the Repair of Spinal Cord Injury. Tissue Eng. Regen. Med..

[18] Jiu J., Liu H.F., Li D.J., Li J.R., Liu L., Yang W.J., Yan L., Li S.Y., Zhang J., Li X.K. (2024). 3D bioprinting approaches for spinal cord injury repair. Biofabrication.

[19] Bedir T., Ulag S., Ustundag C.B., Gunduz O. (2020). 3D bioprinting applications in neural tissue engineering for spinal cord injury repair. Mater. Sci. Eng. C Mater. Biol. Appl..

[20] Szymoniuk M., Mazurek M., Dryla A., Kamieniak P. (2023). The application of 3D-bioprinted scaffolds for neuronal regeneration after traumatic spinal cord injury—A systematic review of preclinical in vivo studies. Exp. Neurol..

[21] Ju D., Dong C. (2024). The combined application of stem cells and three-dimensional bioprinting scaffolds for the repair of spinal cord injury. Neural Regen. Res..

[22] Kačarević Ž.P., Rider P.M., Alkildani S., Retnasingh S., Smeets R., Jung O., Ivanišević Z., Barbeck M. (2018). An Introduction to 3D Bioprinting: Possibilities, Challenges and Future Aspects. Materials.

[23] Yuan T.Y., Zhang J., Yu T., Wu J.P., Liu Q.Y. (2022). 3D Bioprinting for Spinal Cord Injury Repair. Front. Bioeng. Biotechnol..

[24] Gillispie G., Prim P., Copus J., Fisher J., Mikos A.G., Yoo J.J., Atala A., Lee S.J. (2020). Assessment methodologies for extrusion-based bioink printability. Biofabrication.

[25] Panwar A., Tan L.P. (2016). Current Status of Bioinks for Micro-Extrusion-Based 3D Bioprinting. Molecules.

[26] Kumar P., Ebbens S., Zhao X. (2021). Inkjet printing of mammalian cells—Theory and applications. Bioprinting.

[27] Rider P., Kačarević Ž.P., Alkildani S., Retnasingh S., Barbeck M. (2018). Bioprinting of tissue engineering scaffolds. J. Tissue Eng..

[28] Ventura R.D. (2021). An Overview of Laser-assisted Bioprinting (LAB) in Tissue Engineering Applications. Med. Lasers.

[29] Kumar H., Kim K. (2020). Stereolithography 3D Bioprinting. Methods Mol. Biol..

[30] Dell A.C., Wagner G., Own J., Geibel J.P. (2022). 3D Bioprinting Using Hydrogels: Cell Inks and Tissue Engineering Applications. Pharmaceutics.

[31] Zhao L., Zhou Y., Zhang J., Liang H., Chen X., Tan H. (2023). Natural Polymer-Based Hydrogels: From Polymer to Biomedical Applications. Pharmaceutics.

[32] Thang N.H., Chien T.B., Cuong D.X. (2023). Polymer-Based Hydrogels Applied in Drug Delivery: An Overview. Gels.

[33] Jiang J.P., Liu X.Y., Zhao F., Zhu X., Li X.Y., Niu X.G., Yao Z.T., Dai C., Xu H.Y., Ma K. (2020). Three-dimensional bioprinting collagen/silk fibroin scaffold combined with neural stem cells promotes nerve regeneration after spinal cord injury. Neural Regen. Res..

[34] Sun Y., Yang C., Zhu X., Wang J.J., Liu X.Y., Yang X.P., An X.W., Liang J., Dong H.J., Jiang W. (2019). 3D printing collagen/chitosan scaffold ameliorated axon regeneration and neurological recovery after spinal cord injury. J. Biomed. Mater. Res. A.

[35] Wang J., Kong X., Li Q., Li C., Yu H., Ning G., Xiang Z., Liu Y., Feng S. (2021). The spatial arrangement of cells in a 3D-printed biomimetic spinal cord promotes directional differentiation and repairs the motor function after spinal cord injury. Biofabrication.

[36] Liu S., Xie Y.Y., Wang B. (2019). Role and prospects of regenerative biomaterials in the repair of spinal cord injury. Neural Regen. Res..

[37] Liu X.Y., Hao M.M., Chen Z.J., Zhang T., Huang J., Dai J.W., Zhang Z.J. (2021). 3D bioprinted neural tissue constructs for spinal cord injury repair. Biomaterials.

[38] Khaing Z.Z., Milman B.D., Vanscoy J.E., Seidlits S.K., Grill R.J., Schmidt C.E. (2011). High molecular weight hyaluronic acid limits astrocyte activation and scar formation after spinal cord injury. J. Neural Eng..

[39] Joung D., Truong V., Neitzke C.C., Guo S.Z., Walsh P.J., Monat J.R., Meng F.B., Park S.H., Dutton J.R., Parr A.M. (2018). 3D Printed Stem-Cell Derived Neural Progenitors Generate Spinal Cord Scaffolds. Adv. Funct. Mater..

[40] Lee H.Y., Moon S.H., Kang D., Choi E., Yang G.H., Kim K.N., Won J.Y., Yi S. (2023). A multi-channel collagen conduit with aligned Schwann cells and endothelial cells for enhanced neuronal regeneration in spinal cord injury. Biomater. Sci..

[41] Janmey P.A., Winer J.P., Weisel J.W. (2009). Fibrin gels and their clinical and bioengineering applications. J. R. Soc. Interface.

[42] Skardal A., Atala A. (2015). Biomaterials for integration with 3-D bioprinting. Ann. Biomed. Eng..

[43] Gao S., Zhao P., Lin C., Sun Y., Wang Y., Zhou Z., Yang D., Wang X., Xu H., Zhou F. (2014). Differentiation of human adipose-derived stem cells into neuron-like cells which are compatible with photocurable three-dimensional scaffolds. Tissue Eng. Part A.

[44] Mahadik B., Margolis R., McLoughlin S., Melchiorri A., Lee S.J., Yoo J., Atala A., Mikos A.G., Fisher J.P. (2022). An open-source bioink database for microextrusion 3D printing. Biofabrication.

[45] Wang Z., Cui H., Liu M., Grage S.L., Hoffmann M., Sedghamiz E., Wenzel W., Levkin P.A. (2022). Tough, Transparent, 3D-Printable, and Self-Healing Poly(ethylene glycol)-Gel (PEGgel). Adv. Mater..

[46] Roman J.A., Reucroft I., Martin R.A., Hurtado A., Mao H.Q. (2016). Local Release of Paclitaxel from Aligned, Electrospun Microfibers Promotes Axonal Extension. Adv. Healthc. Mater..

[47] Guler S., Eichholz K., Chariyev-Prinz F., Pitacco P., Aydin H.M., Kelly D.J., Vargel İ. (2022). Biofabrication of Poly(glycerol sebacate) Scaffolds Functionalized with a Decellularized Bone Extracellular Matrix for Bone Tissue Engineering. Bioengineering.

[48] dos Santos M.G., Franca F.S., Prestes J.P., Teixeira C., Sommer L.C., Sperling L.E., Pranke P. (2024). Production of a Bioink Containing Decellularized Spinal Cord Tissue for 3D Bioprinting. Tissue Eng. Part A.

[49] Lee S.J., Zhu W., Nowicki M., Lee G., Heo D.N., Kim J., Zuo Y.Y., Zhang L.G. (2018). 3D printing nano conductive multi-walled carbon nanotube scaffolds for nerve regeneration. J. Neural Eng..

[50] Hopley E.L., Salmasi S., Kalaskar D.M., Seifalian A.M. (2014). Carbon nanotubes leading the way forward in new generation 3D tissue engineering. Biotechnol. Adv..

[51] Ahlfeld T., Guduric V., Duin S., Akkineni A.R., Schütz K., Kilian D., Emmermacher J., Cubo-Mateo N., Dani S., Witzleben M.v. (2020). Methylcellulose—A versatile printing material that enables biofabrication of tissue equivalents with high shape fidelity. Biomater. Sci..

[52] Zhang W. (2022). Recent Progress in Bioprinting: From Bioink Design to Applications. Bioengineering.

[53] Tan X.H., Liu L., Mitryashkin A., Wang Y., Goh J.C.H. (2022). Silk Fibroin as a Bioink—A Thematic Review of Functionalization Strategies for Bioprinting Applications. ACS Biomater. Sci. Eng..

[54] Ye D., Wang Q., Yang Y., Chen B., Zhang F., Wang Z., Luan Z. (2023). Identifying Genes that Affect Differentiation of Human Neural Stem Cells and Myelination of Mature Oligodendrocytes. Cell Mol. Neurobiol..

[55] Lee S., Nam H., Joo K.M., Lee S.H. (2022). Advances in Neural Stem Cell Therapy for Spinal Cord Injury: Safety, Efficacy, and Future Perspectives. Neurospine.

[56] Baker E.W., Kinder H.A., West F.D. (2019). Neural stem cell therapy for stroke: A multimechanistic approach to restoring neurological function. Brain Behav..

[57] Gao Y., Wang Y., Wu Y., Liu S. (2024). Biomaterials targeting the microenvironment for spinal cord injury repair: Progression and perspectives. Front. Cell. Neurosci..

[58] Liu S., Yang H., Chen D., Xie Y.Y., Tai C.X., Wang L.D., Wang P., Wang B. (2022). Three-dimensional bioprinting sodium alginate/gelatin scaffold combined with neural stem cells and oligodendrocytes markedly promoting nerve regeneration after spinal cord injury. Regen. Biomater..

[59] Hu Y., Zhang H., Wei H., Cheng H., Cai J., Chen X., Xia L., Wang H., Chai R. (2022). Scaffolds with anisotropic structure for neural tissue engineering. Eng. Regen..

[60] Moradi S., Mahdizadeh H., Šarić T., Kim J., Harati J., Shahsavarani H., Greber B., Moore J.B. (2019). Research and therapy with induced pluripotent stem cells (iPSCs): Social, legal, and ethical considerations. Stem Cell Res. Ther..

[61] Aboul-Soud M.A.M., Alzahrani A.J., Mahmoud A. (2021). Induced Pluripotent Stem Cells (iPSCs)-Roles in Regenerative Therapies, Disease Modelling and Drug Screening. Cells.

[62] Zheng Y., Gallegos C.M., Xue H., Li S., Kim D.H., Zhou H., Xia X., Liu Y., Cao Q. (2022). Transplantation of Human Induced Pluripotent Stem Cell-Derived Neural Progenitor Cells Promotes Forelimb Functional Recovery after Cervical Spinal Cord Injury. Cells.

[63] Ma Y.H., Liang Q.Y., Ding Y., Han I., Zeng X. (2022). Multimodal Repair of Spinal Cord Injury With Mesenchymal Stem Cells. Neurospine.

[64] Deng W.S., Liu X.Y., Ma K., Liang B., Liu Y.F., Wang R.J., Chen X.Y., Zhang S. (2021). Recovery of motor function in rats with complete spinal cord injury following implantation of collagen/silk fibroin scaffold combined with human umbilical cord-mesenchymal stem cells. Rev. Assoc. Medica Bras..

[65] Chen C., Xu H.H., Liu X.Y., Zhang Y.S., Zhong L., Wang Y.W., Xu L., Wei P., Chen Y.X., Liu P. (2022). 3D printed collagen/silk fibroin scaffolds carrying the secretome of human umbilical mesenchymal stem cells ameliorated neurological dysfunction after spinal cord injury in rats. Regen. Biomater..

[66] Li Y., Cao X., Deng W., Yu Q., Sun C., Ma P., Shao F., Yusif M.M., Ge Z., Wang K. (2021). 3D printable Sodium alginate-Matrigel (SA-MA) hydrogel facilitated ectomesenchymal stem cells (EMSCs) neuron differentiation. J. Biomater. Appl..

[67] Liu Z., Lai J., Kong D., Zhao Y., Zhao J., Dai J., Zhang M. (2024). Advances in electroactive bioscaffolds for repairing spinal cord injury. Biomed. Mater..

[68] Qin C., Qi Z., Pan S., Xia P., Kong W., Sun B., Du H., Zhang R., Zhu L., Zhou D. (2023). Advances in Conductive Hydrogel for Spinal Cord Injury Repair and Regeneration. Int. J. Nanomed..

[69] Zhang L., Stauffer W.R., Jane E.P., Sammak P.J., Cui X.T. (2010). Enhanced differentiation of embryonic and neural stem cells to neuronal fates on laminin peptides doped polypyrrole. Macromol. Biosci..

[70] Entezari M., Mozafari M., Bakhtiyari M., Moradi F., Bagher Z., Soleimani M. (2022). Three-dimensional-printed polycaprolactone/polypyrrole conducting scaffolds for differentiation of human olfactory ecto-mesenchymal stem cells into Schwann cell-like phenotypes and promotion of neurite outgrowth. J. Biomed. Mater. Res. A.

[71] Ghasemi-Mobarakeh L., Prabhakaran M.P., Morshed M., Nasr-Esfahani M.H., Baharvand H., Kiani S., Al-Deyab S.S., Ramakrishna S. (2011). Application of conductive polymers, scaffolds and electrical stimulation for nerve tissue engineering. J. Tissue Eng. Regen. Med..

[72] Dixon D.T., Gomillion C.T. (2021). Conductive Scaffolds for Bone Tissue Engineering: Current State and Future Outlook. J. Funct. Biomater..

[73] Gao C., Li Y., Liu X., Huang J., Zhang Z. (2023). 3D bioprinted conductive spinal cord biomimetic scaffolds for promoting neuronal differentiation of neural stem cells and repairing of spinal cord injury. Chem. Eng. J..

[74] Song S., Liu X., Huang J., Zhang Z. (2022). Neural stem cell-laden 3D bioprinting of polyphenol-doped electroconductive hydrogel scaffolds for enhanced neuronal differentiation. Biomater. Adv..

[75] Song S., Li Y., Huang J., Cheng S., Zhang Z. (2023). Inhibited astrocytic differentiation in neural stem cell-laden 3D bioprinted conductive composite hydrogel scaffolds for repair of spinal cord injury. Biomater. Adv..

[76] Kiyotake E.A., Thomas E.E., Homburg H.B., Milton C.K., Smitherman A.D., Donahue N.D., Fung K.M., Wilhelm S., Martin M.D., Detamore M.S. (2022). Conductive and injectable hyaluronic acid/gelatin/gold nanorod hydrogels for enhanced surgical translation and bioprinting. J. Biomed. Mater. Res. A.

[77] Kong W., Zhao Y., Xiaoyu Y., Chen J., Chen Y., Zhao Z., Chen X., Wang F., Fu C. (2023). Combined treatment using novel multifunctional MAu-GelMA hydrogel loaded with neural stem cells and electrical stimulation promotes functional recovery from spinal cord injury. Ceram. Int..

[78] Lin A., Shaaya E., Calvert J.S., Parker S.R., Borton D.A., Fridley J.S. (2022). A Review of Functional Restoration From Spinal Cord Stimulation in Patients With Spinal Cord Injury. Neurospine.

[79] Juckett L., Saffari T.M., Ormseth B., Senger J.L., Moore A.M. (2022). The Effect of Electrical Stimulation on Nerve Regeneration Following Peripheral Nerve Injury. Biomolecules.

[80] Zhao Y., Liang Y., Ding S., Zhang K., Mao H.Q., Yang Y. (2020). Application of conductive PPy/SF composite scaffold and electrical stimulation for neural tissue engineering. Biomaterials.

[81] Yao S., Yang Y., Li C., Yang K., Song X., Li C., Cao Z., Zhao H., Yu X., Wang X. (2024). Axon-like aligned conductive CNT/GelMA hydrogel fibers combined with electrical stimulation for spinal cord injury recovery. Bioact. Mater..

[82] Sun Z., Zhu D., Zhao H., Liu J., He P., Luan X., Hu H., Zhang X., Wei G., Xi Y. (2023). Recent advance in bioactive hydrogels for repairing spinal cord injury: Material design, biofunctional regulation, and applications. J. Nanobiotechnol..

[83] Yu H., Yang S., Li H., Wu R., Lai B., Zheng Q. (2023). Activating Endogenous Neurogenesis for Spinal Cord Injury Repair: Recent Advances and Future Prospects. Neurospine.

[84] Keefe K.M., Sheikh I.S., Smith G.M. (2017). Targeting Neurotrophins to Specific Populations of Neurons: NGF, BDNF, and NT-3 and Their Relevance for Treatment of Spinal Cord Injury. Int. J. Mol. Sci..

[85] Liang J., Deng G., Huang H. (2019). The activation of BDNF reduced inflammation in a spinal cord injury model by TrkB/p38 MAPK signaling. Exp. Ther. Med..

[86] Liu X.Y., Chen C., Xu H.H., Zhang Y.S., Zhong L., Hu N., Jia X.L., Wang Y.W., Zhong K.H., Liu C. (2021). Integrated printed BDNF/collagen/chitosan scaffolds with low temperature extrusion 3D printer accelerated neural regeneration after spinal cord injury. Regen. Biomater..

[87] Lee S.J., Zhu W., Heyburn L., Nowicki M., Harris B., Zhang L.G. (2017). Development of Novel 3-D Printed Scaffolds With Core-Shell Nanoparticles for Nerve Regeneration. IEEE Trans. Biomed. Eng..

[88] Gao B., Yang Q., Zhao X., Jin G., Ma Y., Xu F. (2016). 4D Bioprinting for Biomedical Applications. Trends Biotechnol..

[89] Chiang M.Y., Cheng H.W., Lo Y.C., Wang W.C., Chang S.J., Cheng C.H., Lin Y.C., Lu H.E., Sue M.W., Tsou N.T. (2021). 4D spatiotemporal modulation of biomolecules distribution in anisotropic corrugated microwrinkles via electrically manipulated microcapsules within hierarchical hydrogel for spinal cord regeneration. Biomaterials.

[90] Ong W., Pinese C., Chew S.Y. (2019). Scaffold-mediated sequential drug/gene delivery to promote nerve regeneration and remyelination following traumatic nerve injuries. Adv. Drug Deliv. Rev..

[91] Liu X.Y., Song S.S., Chen Z.J., Gao C., Li Y.X., Luo Y., Huang J., Zhang Z.J. (2022). Release of O-GlcNAc transferase inhibitor promotes neuronal differentiation of neural stem cells in 3D bioprinted supramolecular hydrogel scaffold for spinal cord injury repair. Acta Biomater..

[92] Song S.Q., Zhou J., Wan J.M., Zhao X.C., Li K., Yang C.L., Zheng C.C., Wang L.Q., Tang Y.J., Wang C. (2023). Three-dimensional printing of microfiber- reinforced hydrogel loaded with oxymatrine for treating spinal cord injury. Int. J. Bioprint..

[93] Kalluri R., LeBleu V.S. (2020). The biology, function, and biomedical applications of exosomes. Science.

[94] Poongodi R., Chen Y.L., Yang T.H., Huang Y.H., Yang K.D., Lin H.C., Cheng J.K. (2021). Bio-Scaffolds as Cell or Exosome Carriers for Nerve Injury Repair. Int. J. Mol. Sci..

[95] Shang Z., Liu Z., Han M., Fan H., Lu D., Zhou Z., Wang Z., Li Y., Wang X., Wang B. (2024). Individualized bio-scaffold encapsulating siPTEN-loaded exosomes for promoting neuronal regeneration in spinal cord injury. Compos. Part B Eng..

[96] Jin Z., Na J., Lin X., Jiao R., Liu X., Huang Y. (2024). Plant-derived exosome-like nanovesicles: A novel nanotool for disease therapy. Heliyon.

[97] Wang Q., Liu K., Cao X., Rong W., Shi W., Yu Q., Deng W., Yu J., Xu X. (2024). Plant-derived exosomes extracted from Lycium barbarum L. loaded with isoliquiritigenin to promote spinal cord injury repair based on 3D printed bionic scaffold. Bioeng. Transl. Med..

[98] Ma J., Li J., Wang X., Li M., Teng W., Tao Z., Xie J., Ma Y., Shi Q., Li B. (2023). GDNF-Loaded Polydopamine Nanoparticles-Based Anisotropic Scaffolds Promote Spinal Cord Repair by Modulating Inhibitory Microenvironment. Adv. Healthc. Mater..

[99] Xie Y., Song W., Zhao W., Gao Y., Shang J., Hao P., Yang Z., Duan H., Li X. (2018). Application of the sodium hyaluronate-CNTF scaffolds in repairing adult rat spinal cord injury and facilitating neural network formation. Sci. China Life Sci..

[100] Li G., Zhang B., Sun J.H., Shi L.Y., Huang M.Y., Huang L.J., Lin Z.J., Lin Q.Y., Lai B.Q., Ma Y.H. (2021). An NT-3-releasing bioscaffold supports the formation of TrkC-modified neural stem cell-derived neural network tissue with efficacy in repairing spinal cord injury. Bioact. Mater..

[101] Ai A., Hasanzadeh E., Safshekan F., Astaneh M.E., SalehiNamini M., Naser R., Madani F., Shirian S., Jahromi H.K., Ai J. (2023). Enhanced spinal cord regeneration by gelatin/alginate hydrogel scaffolds containing human endometrial stem cells and curcumin-loaded PLGA nanoparticles in rat. Life Sci..

[102] Lee S., Cho D.C., Han I., Kim K.T. (2022). Curcumin as a Promising Neuroprotective Agent for the Treatment of Spinal Cord Injury: A Review of the Literature. Neurospine.

[103] Fan L., Liu C., Chen X., Zheng L., Zou Y., Wen H., Guan P., Lu F., Luo Y., Tan G. (2022). Exosomes-Loaded Electroconductive Hydrogel Synergistically Promotes Tissue Repair after Spinal Cord Injury via Immunoregulation and Enhancement of Myelinated Axon Growth. Adv. Sci..

[104] Cadena M., Ning L.Q., King A., Hwang B., Jin L.Q., Serpooshan V., Sloan S.A. (2021). 3D Bioprinting of Neural Tissues. Adv. Healthc. Mater..

[105] Krych A.J., Rooney G.E., Chen B., Schermerhorn T.C., Ameenuddin S., Gross L., Moore M.J., Currier B.L., Spinner R.J., Friedman J.A. (2009). Relationship between scaffold channel diameter and number of regenerating axons in the transected rat spinal cord. Acta Biomater..

[106] Joung D., Lavoie N.S., Guo S.Z., Park S.H., Parr A.M., McAlpine M.C. (2020). 3D Printed Neural Regeneration Devices. Adv. Funct. Mater..

[107] Koffler J., Zhu W., Qu X., Platoshyn O., Dulin J.N., Brock J., Graham L., Lu P., Sakamoto J., Marsala M. (2019). Biomimetic 3D-printed scaffolds for spinal cord injury repair. Nat. Med..

[108] Nazeri N., Derakhshan M.A., Mansoori K., Ghanbari H. (2022). Improvement of sciatic nerve regeneration by multichannel nanofibrous membrane-embedded electro-conductive conduits functionalized with laminin. J. Mater. Sci. Mater. Med..

[109] Sun X., Zhang C., Xu J., Zhai H., Liu S., Xu Y., Hu Y., Long H., Bai Y., Quan D. (2020). Neurotrophin-3-Loaded Multichannel Nanofibrous Scaffolds Promoted Anti-Inflammation, Neuronal Differentiation, and Functional Recovery after Spinal Cord Injury. ACS Biomater. Sci. Eng..

[110] Huang L., Gao J., Wang H., Xia B., Yang Y., Xu F., Zheng X., Huang J., Luo Z. (2020). Fabrication of 3D Scaffolds Displaying Biochemical Gradients along Longitudinally Oriented Microchannels for Neural Tissue Engineering. ACS Appl. Mater. Interfaces.

[111] Yang J., Yang K., Man W., Zheng J., Cao Z., Yang C.-Y., Kim K., Yang S., Hou Z., Wang G. (2023). 3D bio-printed living nerve-like fibers refine the ecological niche for long-distance spinal cord injury regeneration. Bioact. Mater..

[112] Li Y.X., Cheng S.N., Wen H.L., Xiao L.Y., Deng Z.W., Huang J., Zhang Z.J. (2023). Coaxial 3D printing of hierarchical structured hydrogel scaffolds for on-demand repair of spinal cord injury. Acta Biomater..

[113] Roolfs L., Hubertus V., Spinnen J., Shopperly L.K., Fehlings M.G., Vajkoczy P. (2022). Therapeutic Approaches Targeting Vascular Repair After Experimental Spinal Cord Injury: A Systematic Review of the Literature. Neurospine.

[114] Zhang L.G., Leong K., Fisher J.P. (2022). 3D Bioprinting and Nanotechnology in Tissue Engineering and Regenerative Medicine.

